# Endometrial Adenocarcinoma in Young Women: A Case Report and Review of Literature

**DOI:** 10.7759/cureus.45287

**Published:** 2023-09-15

**Authors:** Jessica Satei, Ariana N Afrakhteh, Kim Abbegail T Aldecoa

**Affiliations:** 1 Medicine, Newcastle University Medical School, Newcastle, GBR; 2 Medicine, Wayne State University School of Medicine, Detroit, USA; 3 Internal Medicine, Trinity Health Oakland Hospital/Wayne State University Program, Pontiac, USA

**Keywords:** female fertility, figo, endometrial endometrioid adenocarcinoma, women’s health, endometrial ca

## Abstract

Endometrial cancer in young women presents a unique challenge to care teams. With over 90% of cases diagnosed in women over the age of 50, its diagnosis can be delayed in younger patients if the medical team does not maintain a high enough index of suspicion. Once diagnosed, treatment options depend on a desire to maintain fertility. We present a case of a 36-year-old female who, following cross-sectional imaging and pathological analysis, was diagnosed with endometrioid endometrial adenocarcinoma. This case explores the epidemiology of endometrial cancer in young women and the importance of a multi-disciplinary approach to the diagnosis and treatment of this rare malignancy.

## Introduction

Endometrial cancer is the most prevalent gynecological malignancy, affecting approximately 3% of American women [1]. The median age at diagnosis is 61 years, with over 90% of cases diagnosed after the age of 50. Endometrial cancer rarely occurs in young women, typically defined as occurring before the age of 50 or menopause, with a small minority of these rare cases occurring before the age of 40. One study that investigated cases of endometrial cancer in women aged below 60 years old found that only 18.7% of these cases occurred in women under the age of 40 [2]. The significant morbidity carried by endometrial cancer is confounded by obstacles to its diagnosis secondary to the low prevalence of the disease in this age group. In addition, unique considerations including preservation of fertility must be made when considering management options, making endometrial cancer in young women a challenge for all involved specialties.

## Case presentation

History and examination

A 36-year-old nulliparous Caucasian female presented with a three-year history of severe dysmenorrhea and menorrhagia which had worsened in the preceding three months. Her past medical history was non-contributory. The patient endorsed an irregular menstrual cycle, heavy flow, and pain localized to the bilateral lower abdomen. Physical examination revealed an area of firmness in the lower left abdominal quadrant associated with pain on palpation. The patient’s body mass index was 28.8. 

Laboratory investigations on admission revealed low hemoglobin (5.9 g/dL), low hematocrit (22.8%), mean corpuscular volume (71.7 FL), and elevated white blood count (12.7 K/mcL). The patient was admitted for further evaluation.

Imaging findings

**Figure 1:**
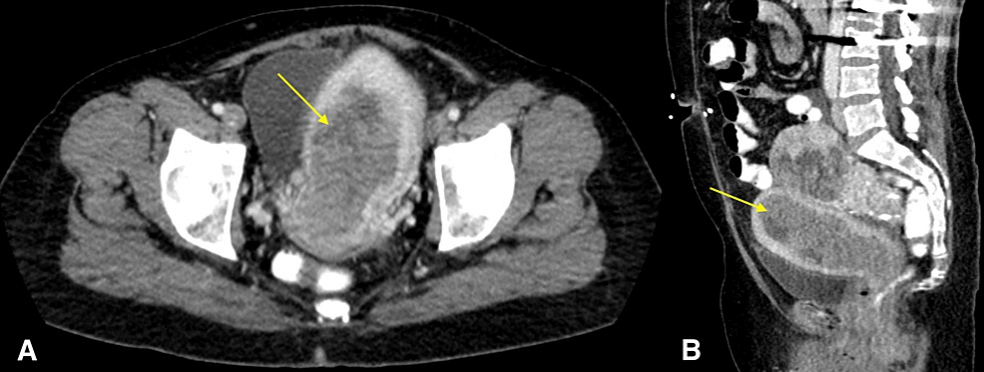
Computed tomography of the abdomen and pelvis with IV contrast in axial (A) and sagittal (B) views. There is marked lobular heterogeneous thickening of the central endometrium measuring up to 5.6 cm (yellow arrow). This mass extends from the uterine fundus to the endocervical canal and upper vagina.

The patient underwent computed tomography (CT) of the abdomen and pelvis with intravenous contrast (Figures 1A, 1B). Examination revealed a marked abnormality of the endometrium, which appeared heterogeneous and measured 5.6 cm in maximal thickness. The mass extended into the endocervical canal and upper vagina, with possible extension into the overlying myometrium. Additionally, several borderline enlarged lymph nodes throughout the abdomen and pelvis measured up to 0.9 cm in short axis, with mild haziness and discrete soft tissue nodularity of the anterior peritoneum. Incidentally, an adjacent large heterogeneous partially calcified mass within the pelvis which measured up to 12.5 cm was visualized, likely representing a uterine leiomyoma. Overall, imaging findings were suspicious for endometrial carcinoma. Additionally, the soft tissue nodularity of the anterior peritoneum raised concerns for peritoneal carcinomatosis.

Histological findings

**Figure 2:**
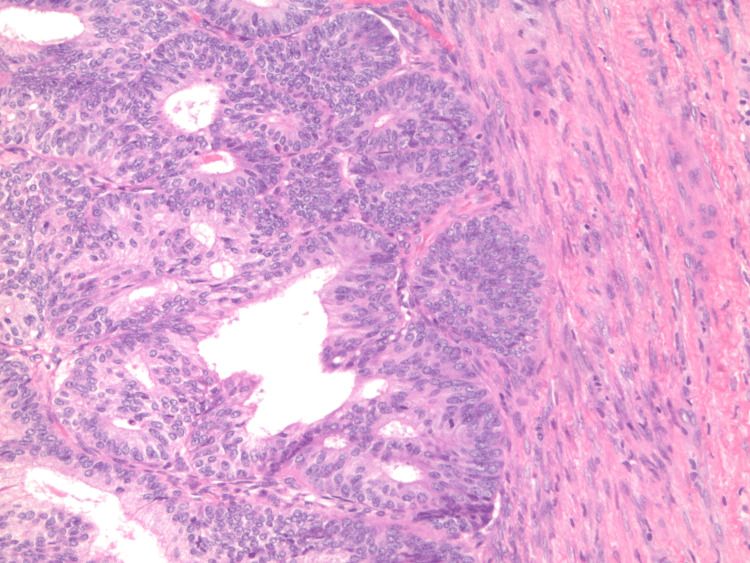
100x H&E stain demonstrating invasion of the myometrium. H&E: hematoxylin and eosin

The patient was referred to general surgery where she underwent total abdominal hysterectomy, bilateral salpingectomy and oophorectomy, and pelvic and para-aortic lymph node sampling. Histological analysis of the uterus demonstrated endometrial endometrioid adenocarcinoma, FIGO grade 3, with invasion of the outer half of the myometrium and cervical stroma (Figure 2). There was evidence of extensive angiolymphatic invasion. Surgical margins were negative. The biopsied nodes in the para-aortic and bilateral pelvic regions were negative for carcinoma. She was not found to have synchronous ovarian cancer.

Following surgery, the patient recovered successfully. She was discharged from the hospital and underwent whole pelvic brachytherapy on an outpatient basis without adjuvant chemotherapy due to the early stage of her endometrial cancer. Currently, the patient is asymptomatic and is being observed. Future care can involve follow-up cross-sectional imaging and monitoring of tumor markers to evaluate for recurrence.

## Discussion

Epidemiology

Two different subtypes of endometrial cancer are recognized. Type I endometrial cancer, known as endometrioid endometrial carcinoma, occurs in approximately 80% of cases, is secondary to hyperestrogenism, and is typically seen in patients aged 55-65 years old [3]. Type II endometrial cancer, known as non-endometrioid endometrial carcinoma, comprises the other 20% of cases, occurs secondary to endometrial atrophy, and typically affects women aged 65-75 years old [3]. Our case involves endometrioid endometrial carcinoma (type I endometrial cancer).

Risk factors for young women to develop endometrial cancer include obesity, smoking, higher insulin levels, type II diabetes, hypertension, and early menarche. A BMI of >30 is typically considered the greatest single individual risk factor for early-onset endometrial cancer. Additionally, it is associated with Lynch syndrome and polycystic ovarian syndrome [2,4]. Our patient was not known to have any of the above risk factors.

Additionally, patient race has been found to be associated with the young onset of endometrial cancer, with young black women more likely to be diagnosed with endometrial carcinoma. Furthermore, tissue sampling more commonly demonstrates poorly differentiated and non-endometrioid subtypes in black women. Five-year survival rates are significantly better in young white women compared to their black counterparts. For example, in cases of stage IB tumors, the five-year survival rate is 90.6% and 81.5%, respectively, while in cases of stage III cancer, the five-year survival rate is 75.1% and 63.3%, respectively [5]. Ultimately, young black women have a worse prognosis when diagnosed with endometrial carcinoma due to a combination of higher rates of subtypes associated with poor outcomes and a higher rate of endometrtial cancer overall.

Investigation into the possibility of endometrial carcinoma more often occurs secondary to abnormal vaginal bleeding. In postmenopausal women, this is more easily identifiable. In premenopausal women, abnormal menstrual cycles with long or heavy bleeding may prompt further evaluation. Additionally, vaginal discharge, lower abdominal or pelvic pain, dyspareunia, or dysuria may be present. Interestingly, some young women with endometrial cancer are incidentally encountered during hysteroscopic examinations during reproductive procedures [4].

Radiological diagnosis

In most cases, when endometrial cancer is suspected, initial radiological investigation involves transvaginal and transabdominal ultrasound. Transvaginal ultrasonography is especially useful for the evaluation of the endometrium including measurement of endometrial thickness. In postmenopausal women, an endometrial thickness of greater than 5 mm, or greater than 8 mm if on tamoxifen or hormone replacement therapy, should prompt tissue sampling [6].

In premenopausal women, endometrial thickness can vary depending on the patient's menstrual cycle. Endometrial thickness is greatest in the secretory and preovulatory phases, and the least during menstruation. Typically, endometrial thickness does not exceed 16 mm in premenopausal women, regardless of the phase of the menstrual cycle. However, there can be significant individual variability in this number, further confounded by irregular cycles and unknown or unreliable self-reporting of a patient’s last menstrual period. In cases where the radiologist is unsure of a thickened endometrium, other sonographic signs can be utilized including a heterogeneous and irregular appearing endometrium, with evidence of a mass or fluid collection [6]. Further sonographic imaging can be obtained 10-14 days following an indeterminate ultrasound examination during a different phase of the menstrual cycle.

Following ultrasound, further radiological examinations include cross-sectional imaging using CT or magnetic resonance imaging (MRI). Contrast-enhanced CT is preferred for assessing for evidence of metastasis, however, is limited in evaluating endometrial thickening or a mass in early cases. Contrast-enhanced MRI pelvic mass protocol can be obtained to evaluate for local extension within the pelvis, including further evaluation of the endometrium, as well as invasion of the cervical stroma and myometrium [7]. MRI is preferred when determining FIGO stage due to improved visualization of the myometrial invasion compared to CT. Nuclear imaging modalities, including PET/CT, are not typically employed, although may provide further evaluation for distant metastasis in advanced cases [8].

Pathology diagnosis

Microscopic evaluation of endometrial carcinoma will demonstrate confluent glands lacking stroma with cribriform configurations. Proliferative type endometrium is visualized with cellular and nuclear enlargement [9,10]. Pathology reports typically comment on tumor grade, myometrial, serosal, lymphovascular, cervical stromal, ovarian, and fallopian tube invasion.

Histological FIGO staging is used based on the architecture of the tumor, depending on the extent of solid non-glandular nonsquamous growth (Table 1) [11]. FIGO stages I-II have better prognosis compared to FIGO stage III, with FIGO stage III tumors considered high grade. Our patient’s case was deemed FIGO stage III on histopathological analysis.

**Table 1:** FIGO histopathologic grading system for endometrial carcinoma.

Grade	Architecture
1	≤5% solid non-glandular, non-squamous growth
2	6%-50% solid non-glandular, non-squamous growth
3	>50% solid, non-glandular non-squamous growth

Endometrioid endometrial subtypes are associated with PTEN gene mutations [12]. Other altered genes in type I cancer include KRAS and CTNNB1 (beta-catenin). Non-endometrioid endometrial subtypes are associated with p53 mutations and carry a poorer prognosis [12]. Other immunohistochemical changes in type II carcinoma involve cyclin E and p16.

Synchronous ovarian cancer is seen in a significantly higher number of young women with endometrial cancer below the age of 40 compared to their 41-60-year-old counterparts, 9.2% versus 0.7%, respectively [2]. Our patient was not found to have synchronous ovarian cancer.

Treatment

Treatment for endometrial carcinoma involves FIGO staging. Table 2 outlines the FIGO staging system for uterine carcinoma [13]. Our patient was staged as a FIGO stage II secondary to cervical stromal invasion without extension beyond the uterus.

**Table 2:** FIGO staging system utilized for treatment of uterine cancer.

Stage	Description
IA	Tumor confined to the uterus involving <50% invasion of the myometrium
IB	Tumor confined to the uterus involving ≥50% invasion of the myometrium
II	Tumor invades the cervical stroma, without extending beyond the uterus
IIIA	Local of regional spread of tumor involving serosa or adnexa
IIIB	Local or regional spread of tumor involving vagina or parametrium
IIIC	Local or regional spread of tumor involving pelvic or paraaortic lymph nodes
IVA	Invasion of the pelvic wall, lower one-third of the vagina, or hydronephrosis/non-functioning kidney with invasion of the bladder or bowel mucosa
IVB	Invasion of the pelvic wall, lower one-third of the vagina, or hydronephrosis/non-functioning kidney with extrapelvic and distant metastasis, including abdomen or inguinal lymph nodes

Typically, surgical treatment for endometrial cancer involves hysterectomy with bilateral salpingo-oophorectomy with or without lymphadenectomy. Adjuvant chemotherapy and/or radiotherapy are typically employed, with a combination of paclitaxel, doxorubicin, and a platin used [14]. When employed, genomic and molecular analysis can identify more efficient and individualized regimens [15].

In young women with endometrial carcinoma, fertility-sparing treatments are often sought. However, literature has shown limited efficacy of conservative treatment. Uterine preservation is typically only considered in cases of FIGO stage IA endometrial cancer [4]. Typically, this can be done in conjunction with high dose progestogen treatment, such as the use of systemic medroxyprogesterone acetate or levonorgestrel intrauterine systems [16]. These would be used for 6-12 months, with complete remission occurring in 80%-90% of patients. Metformin has also been employed in obese patients due to metformin's effects on insulin resistance and weight loss [17].

A meta-analysis of women who underwent fertility-sparing treatment for atypical hyperplasia and endometrial cancer found a 12-month remission of 78.0%, 12-month recurrence of 9.6%, with 32% of subjects achieving one or more successful pregnancy [18]. In our patient's case, invasion of the cervical stroma made conservative treatment implausible.

Follow-up for endometrial cancer involves follow-up cross-sectional imaging including CT, MRI, or PET/CT. Additionally, the tumor marker cancer antigen 125 (CA-125) can be obtained at the time of diagnosis and following treatment to evaluate for recurrence. Typically, this tumor marker is obtained in cases of advanced endometrial cancer at the time of diagnosis, such as cases with lymph node metastasis, extra-uterine disease, or deep myometrial invasion [19]. This tumor marker was not obtained in our case. Approximately 4-20% of patients with endometrial cancer develop regional recurrence, with higher rates of recurrence in patients with locally advanced disease initially and most cases occurring within the first two years after treatment [20].

## Conclusions

Endometrial adenocarcinoma in young women is a rare and devastating diagnosis. Despite making a small proportion of all endometrial cancer cases, clinical teams should maintain an appropriate degree of clinical suspicion for endometrial cancer, especially for patients who have known risk factors or associated syndromes. Further evaluation with cross-sectional imaging and pathologic analysis can help in making this diagnosis. Ultimately, while fertility-sparing treatments are an attractive alternative to surgery resulting in permanent loss of fertility, these can unfortunately only be employed in a limited subset of cases.
